# Use of ultrasonography in developmental dysplasia of the hip

**DOI:** 10.1007/s11832-014-0561-8

**Published:** 2014-02-09

**Authors:** Hakan Ömeroğlu

**Affiliations:** Section of Pediatric Orthopaedics, Department of Orthopaedics and Traumatology, Faculty of Medicine, Eskişehir Osmangazi University Hospital, 26480 Eskisehir, Turkey

**Keywords:** Ultrasonography, Developmental dysplasia of the hip, Newborn hip screening

## Abstract

**Purpose:**

Ultrasonography has been used as a diagnostic tool in developmental dysplasia of the hip (DDH) during early infancy since the early 1980s. The aim of this review article is to summarise the technique, benefits and shortcomings of four infantile hip ultrasonography methods, focusing mainly on the Graf method, and to assess the effectiveness of ultrasonographic newborn hip screening programmes.

**Methods:**

Several infantile hip ultrasonography methods have been defined to assess the relationship between the femoral head and acetabulum. The Graf, Harcke, Terjesen and Suzuki methods are the universally known ones. The Graf method is composed of a quantitative classification system, while the Harcke and Suzuki methods have qualitative definitions and the Terjesen method contains both quantitative and qualitative descriptions.

**Results:**

Although the results of several studies assessing the sensitivity and consistency of the ultrasonography methods have still not proven a clear dominance of one of these techniques, the primary advantage of the Graf method is that it has a standardised examination technique, as well as a very well defined numeric hip typing system. The importance of newborn hip screening has been universally accepted, but there is still no strong evidence regarding the superiority of either universal (screening of all newborns) or selective (screening of high-risk newborns) ultrasonographic newborn hip screening programmes.

**Conclusions:**

An effective ultrasonographic method should include simple, precise, quantitative and consistent definitions for a proper examination and diagnosis. Both universal and selective ultrasonographic newborn hip screening programmes have significantly decreased the rate of late detected DDH and lessened the need for surgical treatment.

## Introduction

Plain radiography was the gold standard for the radiological diagnosis of developmental dysplasia of the hip (DDH) in all age groups up to the early 1980s. However, exposure to radiation and difficulties in the precise anatomical definition of the relationship between the cartilage femoral head and the cartilage and bony acetabular roofs were the two main disadvantages of the use of plain radiography during early infancy in DDH. Reinhard Graf from Stolzalpe, Austria developed his technique in the late 1970s and published his initial experiences concerning the use of hip ultrasonography for the early radiological diagnosis of DDH in the early 1980s [1]. It is possible to make a multiplanar examination and to determine the position of the femoral head with respect to the acetabulum by using a real-time ultrasonography [2]. Ultrasonography can detect the hip problems that can be missed by clinical and radiographic examinations [2, 3]. As early and accurate diagnosis of DDH is believed to be the most important point for satisfactory treatment, hip ultrasonography has become the most commonly used diagnostic tool for DDH during early infancy, and for many years.

The aim of this review article is to summarise the procedural details, advantages and disadvantages of the four well-known infantile hip ultrasonography methods (Graf, Harcke, Terjesen and Suzuki methods), focusing mostly on the Graf method, as well as to review the medical aspects of ultrasonographic newborn hip screening programmes.

## Graf method

The infantile hip ultrasonography method of Graf is the one that was defined first and is perhaps the most widely used. If the previously well-defined examination, interpretation and measurement techniques are meticulously followed, it is easy to manage the newborn hip problem by using this method [4].

**Fig. 1:**
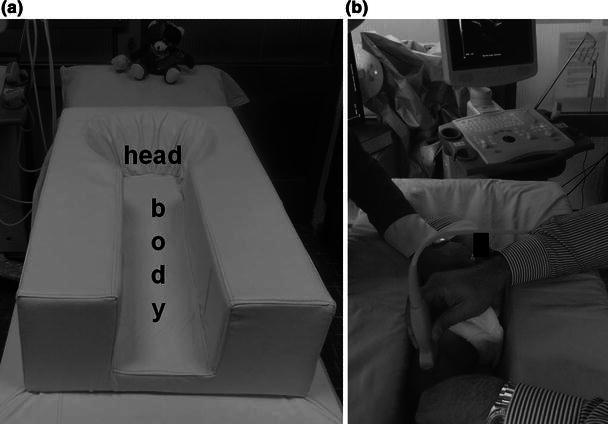
**a** The special positioning apparatus that maintains the baby in the lateral decubitus position. **b** Ultrasonographic examination of the right hip of a baby in the special positioning apparatus

**Fig. 2:**
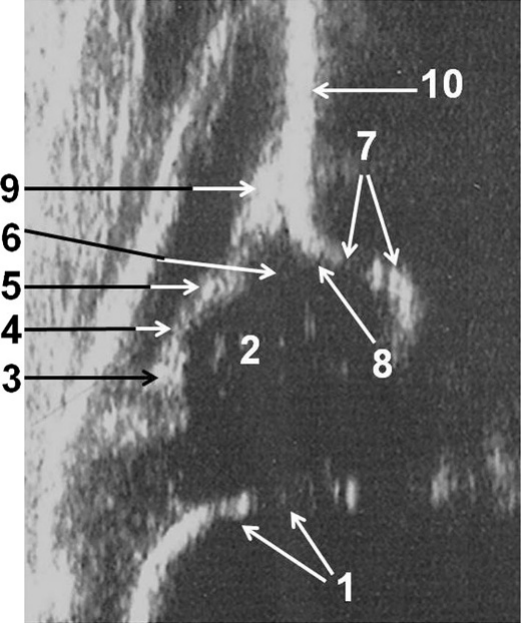
Anatomical identification of the structures in an infantile hip sonogram in the frontal plane at rest: *1* chondro-osseous junction, *2* femoral head, *3* synovial fold, *4* joint capsule, *5* acetabular labrum, *6* cartilage roof, *7* lower limb of the ilium and bony roof, *8* bony rim (the point where the concavity of the bony acetabular roof changes to the convexity of the iliac bone or the most lateral point of the acoustic shadow in the bony acetabular roof), *9* perichondrium, *10* iliac bone

**Fig. 3:**
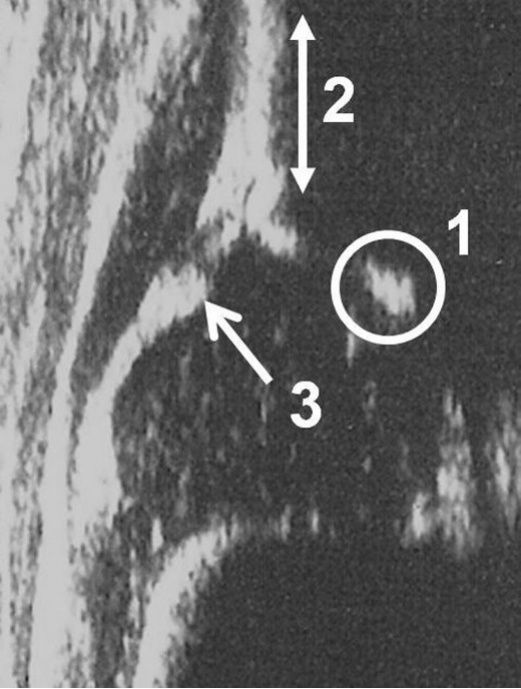
Standard plane for the Graf method [4]. *1* The lower limb of the ilium is clearly visible. This means that the sectional plane passes through the centre of the acetabulum. *2* A straight iliac wing silhouette exists. This means that the probe is parallel to the iliac bone. *3* The labrum is clearly visible

The Graf method should be performed by using a linear array probe in the lateral decubitus position that is maintained by a cradle [4] (Fig. 1a, b). In addition, a probe-guiding system is recommended to avoid tilting effects [4]. The sonogram reflects the position of the resting hip joint in the frontal plane and the anatomical landmarks have been clearly defined. Before starting to classify the hip joint, it is essential to identify the eight anatomical landmarks; chondro-osseous junction, femoral head, synovial fold, hip joint capsule, acetabular labrum, acetabular hyaline cartilage, acetabular bony roof and acetabular bony rim (Fig. 2), as well as to check the usability of the sonogram. The usability check includes the assessment of a sonogram as to whether or not it has a standard plane. If a sonogram contains a clearly visible lower limb of the bony ilium in the depth of the acetabular fossa, as well as an apparent acetabular labrum and a straight iliac wing contour, this means that it has a standard plane [4] (Fig. 3). If the anatomical identification cannot be made or the standard plane is missing in a sonogram, it is of no value and must not be used for diagnosis. The only exception is in dislocated hips. In such hips, non-standard sonograms can be used for the evaluation but not for the measurement, as the superior, lateral and posterior displacement of the femoral head prevents the visualisation of the femoral head and the centre of the acetabulum in the same frontal section [4]. In older children, a large femoral head ossification centre can obscure the visualisation of the lower limb, which is essential for obtaining a standard plane, so this method is ultimately limited by the age of the patient [4]. However, the Graf method may be used in older children if the visualisation problem of the lower limb can be overcome [5].

**Fig. 4:**
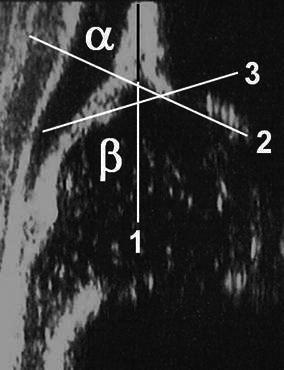
Measurement of the angles in the Graf method [4]. *1* The base line starts from the uppermost point of the proximal perichondrium and is drawn caudally tangential to the iliac bone. *2* The bony roof line starts from the inferior border of the lower limb and is drawn tangentially to the bony roof. *3* The cartilage roof line is drawn between the bony rim and the centre of the labrum. The *α* angle is measured between lines *1* and *2*. The *β* angle is measured between lines *1* and *3*

According to the Graf ultrasonographic hip classification system, the *α* and *β* angles are the quantitative indicators of the bony and cartilage acetabular roofs, respectively (Fig. 4). The *α* angle mainly determines the hip type and the other parameters, such as the age of the patient, *β* angle value, *β* angle value under stress, course of the perichondrium of the cartilage acetabular roof and structural changes in the cartilage roof, give particular differentiations [4] (Table 1). A hip joint becomes ultrasonographically mature at 34 weeks of gestation [6]. If an initially mature (type I) hip deteriorates over time, it is due to a neuromuscular hip instability, a hip joint effusion or a secondary hip dysplasia following a successful treatment. Otherwise, the initial diagnosis is wrong [4, 7]. Graf advocates the immediate treatment of type IIa− and worse hips [4]. However, there still exists controversy in the natural history and management of immature hips. Graf type IIa hips have a lower spontaneous normalisation rate and a higher treatment rate in girls than in boys [8]. Graf recommends to treat the type IIa− hips for completely avoiding the development of residual hip dysplasia and to closely follow the type IIa+ hips for determining whether or not a mature hip can be attained by the end of 3 months [4, 7]. Besides, nearly one in every four type IIb hips carries the risk of development of residual hip dysplasia in the long-term follow-up, even if they have initially been treated with success [9].

**Table 1:** Ultrasonographic hip types according to the Graf method [4]

Hip type	Description	Bony roof	Bony rim	Cartilage roof	*α* angle	*β* angle	Subtype
Type I	Mature hip	Good	Angular/blunt	Covers the femoral head	≥60°	<77°	Ia: *β* ≤ 55° Ib: *β* > 55°
Type IIa	Physiologically immature (age ≤3 months)	Deficient	Rounded	Covers the femoral head	50°–59°	>55°	IIa+: *α* = 55°–59° (at 6 weeks of age) IIa−: *α* = 50°–54° (at 6 weeks of age)
Type IIb	Delay of ossification (age >3 months)	Deficient	Rounded	Covers the femoral head	50°–59°	>55°	
Type IIc	Critical hip	Severely deficient	Rounded to flattened	Still covers the femoral head	43°–49°	<77°	IIc stable: under pressure *β* < 77° IIc unstable: under pressure *β* > 77°
Type D	Decentring hip	Severely deficient	Rounded to flattened	Displaced	43°–49°	>77°	
Type III	Dislocated hip	Poor	Flattened	Pressed upwards, perichondrium slopes cranially	<43°		IIIa: hypoechoic cartilage acetabular roof IIIb: hyperechoic cartilage acetabular roof
Type IV	Dislocated hip	Poor	Flattened	Pressed downwards, perichondrium is horizontal or dips caudally	<43°		

## Harcke method

This method was initially described by Theodore Harcke and associates from Wilmington, DE, USA in 1984 [10]. The ultrasonographic examination of the hip by this method is performed using a linear probe by the lateral approach while the patient is positioned supine or lateral decubitus [11]. Several frontal and transverse images of the hip at rest and in stress are obtained by placing the probe in different positions. The previously defined views of the Harcke method are as follows [10–12]:

1. Frontal neutral view: frontal section of the hip joint in the neutral position.
2. Frontal flexion view: frontal section of the hip joint in 90° flexion.
3. Transverse neutral view: transverse section of the hip joint in the neutral position.
4. Transverse flexion view: transverse section of the hip joint in 90° flexion.

Evaluation of the combination of two views in perpendicular planes is essential for making the diagnosis. View 1 in the mid-acetabular plane at rest plus view 4 with and without stress are used [3, 10–12]. This recommendation is also accepted by the medical associations [13]. So, it is possible to assess the position, stability and morphology of the hip joint. The qualitative description of the sonogram as normal, subluxated, slightly dislocated or dislocated is made after assessing the two previously mentioned perpendicular view components.

The same institutional team subsequently defined the bony rim percentage (BRP), which was an expression of the relative coverage of the femoral head by the bony acetabulum in the frontal flexion view [14]. However, the usability of the coverage percentage seems to be open to discussion, as the coverage of the ellipsoid femoral head can alter with rotation of the femoral head in a frontal plane image. Validation using the quantitative measurements such as Graf’s *α* angle or the BRP (currently named as the femoral head coverage) is optional in the Harcke method [11].

Harcke et al. [12] regarded this method to be reliable and accurate for the early diagnosis of DDH.

## Terjesen method

**Fig. 5:**
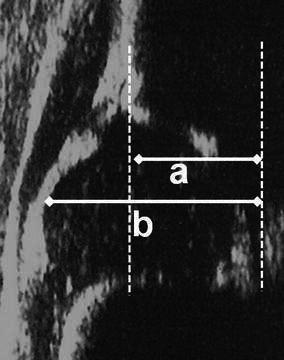
Measurement of the femoral head cover (FHC) [18], where *a* is the distance between the acetabular fossa and the bony rim, and *b* is the distance between the acetabular fossa and the lateral joint capsule. FHC = *a*/*b* × 100

**Fig. 6:**
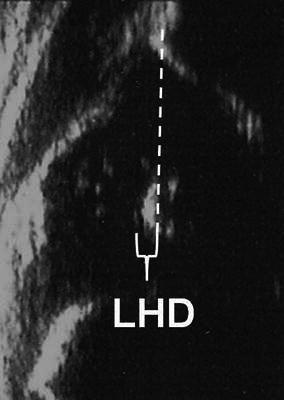
Measurement of the lateral head distance (LHD) [16], which is the distance between the lateral tangent of the femoral head ossification centre and the bony rim

This method was initially defined by Terje Terjesen and associates from Oslo, Norway in the late 1980s [15, 16]. The examination is performed by the lateral approach with the patient in the supine position, and the type of the probe is either linear or sector [15–17]. Static and dynamic scanning in the frontal and transverse planes are performed [15]. This method includes numeric measurements as well as qualitative descriptions. The femoral head cover (FHC) is the coverage percentage of the entire cartilaginous femoral head by the acetabular bony roof (Fig. 5). The lower normal limit for the FHC in infants older than 1 month of age is 50 %, but this parameter can only be used in the first year of life, as the acetabular fossa cannot be visualised due to the obstruction of the ossified femoral head after that age [18]. In patients where the femoral head ossification centre appears, the lateral head distance (LHD), which is an expression of the uncovered part of the ossific nucleus, is measured and it can have a minus sign in normal hips [16, 17] (Fig. 6). However, the use of ossific nucleus as a landmark in a sonogram can be questionable in especially younger infants, as the ossific nucleus is not always located in the center of the cartilaginous femoral head. The upper normal limit for this parameter is 2–3 mm under 12 months of age, 3–4 mm at age 1–2 years, 4 mm at age 2–3 years, 5 mm at 4–7 years, 6 mm at 8–11 years and 7 mm over 11 years of age [18, 19]. In addition to these measurements, the shape of the lateral bony rim is defined (normal, defective or rounded) and the *α* angle of Graf is measured, if possible [16]. All these parameters provide the basis for classification as normal, dysplasia, subluxation or dislocation. This method can be used from birth to the adolescent period [15–17].

## Suzuki method

This method was initially defined by Shiego Suzuki and associates from Shiga, Japan in the early 1990s and includes the simultaneous examination of both hips using a large linear probe by an anterior approach [20]. A standard plane, showing both pubic bones and femoral heads, is obtained by placing the large linear probe on the pubis while the patient lies supine with the hips extended. Two lines are drawn to make the diagnosis on the image. *P* is the line drawn along the anterior surface of the pubic bones and *E* is the line perpendicular to line *P* drawn from the lateral margins of both pubic bones [20]. In a normal hip, the femoral head lies behind the *P* line and intersects the *E* line medially. In a slight dislocation, there exists a gap between the femoral head and the *E* line. In a high dislocation, the femoral head crosses the *P* line and the maximum diameter of the femoral head cannot be seen on the sonogram [20]. When a dislocation is detected, an examination with the hips flexed and abducted is made. The metaphysis of the femur is used to identify the femoral head in an abducted and flexed hip. It was reported that examining both hips in an abduction brace or a plaster cast was possible with this method [20]. Suzuki et al. [20] noted that the diagnostic results obtained by this method was comparable with those by the Graf method.

It can be emphasised that the previously mentioned methods, except the Graf method, include mostly qualitative definitions as well as even the ultrasonographic examination of the baby, which does not have any standardisation. Besides, some of the previously described methods need quantitative measurements of the Graf method, but the Graf method does not need any additional examination methods. In addition, in the static method of Graf, even the ultrasonographic examination of the baby has clearly been described, as well as every hip type having very clear definition based on numeric measurements. So, the observer dependency of this method seems to be negligible if it is performed correctly, as every step of the method has been very well standardised.

## Consistency of the infantile hip ultrasonography methods

Several studies concerning the intraobserver and interobserver agreements of the methods were published, and most of them were focused on the Graf method. The reported intraobserver and interobserver reliability concerning the hip typing in the Graf method ranged from moderate to substantial and from fair to substantial, respectively [21–26]. Besides, the reported intraobserver and interobserver measurement variability of the *α* angle ranged from 4° to 11° and from 3° to 13°, respectively [21, 23, 24, 26, 27]. The reported intraobserver and interobserver measurement variability of the *β* angle was between 6° and 14° and between 6° and 19°, respectively [23, 24, 26, 27].

Terjesen et al. [16, 17] reported that a good level of reproducibility of the quantitative parameters of the Terjesen method could be obtained.

The results of three different studies revealed that it was better to measure the *α* and *β* angles than to compute the FHC for precisely defining the hip morphology and managing the hip pathology [24, 28, 29].

In studies comparing the sensitivity and reliability of the Graf and Terjesen methods in hip typing, different conclusions were obtained. In one study, a nearly three times lower rate of pathological hip diagnosis and a slightly better intraobserver reliability in the Graf method than in the Terjesen method was reported [22]. In another study, a lower rate of dislocation, subluxation or possible dysplasia diagnosis and a better interobserver agreement in the Terjesen method than in the Graf method was noted [30].

In a study comparing the Graf, Harcke and Suzuki methods, a correlation was found between the three methods in normal and dislocated hips; however, Graf type IIa and IIb hips were commonly considered normal when the same hips were assessed by using the other two methods [31].

## Medical aspects of ultrasonographic newborn hip screening

It was previously reported that DDH was the underlying aetiology in about 25 % of the performed hip replacements under the age of 40 years and the initial diagnosis age ranged from 0 to 39 years, with a mean of 8 years in these patients [32]. This significant finding again emphasises the importance of newborn hip screening. However, to date, there has been no prospective controlled clinical study comparing the benefits of hip screening and early treatment with no hip screening and late treatment [33]. The main purpose of a newborn hip screening programme is to detect DDH as early as possible, so that early treatment can be give and the need for surgical treatment as well as the development of residual hip dysplasia can be avoided [34]. A recent decision analytic model emphasised the importance of clinical and ultrasonographic newborn hip screening for avoiding late degenerative hip arthritis [35]. There still exists controversy concerning the methodology in newborn hip screening programmes. There are still two important debates to be clearly enlightened [34]. Can clinical screening alone still be sufficient to detect DDH in all newborns? If not, is ultrasonographic screening needed for all newborns (universal hip screening) or only for high-risk babies (selective hip screening)?

The answer to the first question seems to depend on the accuracy of the clinical examination as well as the level of experience of the examiners. It was previously reported that the clinical examination of a dedicated and experienced clinician could still detect almost all pathological hips [36]. In contrast, the risk of missing the entire pathological hips by clinical screening only was reported to be 2.6/1,000 and half of them were subluxated or dislocated hips [37]. Nevertheless, the radiographic findings of the only clinical screening and ultrasonographic screening groups were found to be similar at maturity [38]. It has been our own experience that Graf type IIc and worse hips can be detected by clinical examination in experienced hands, but the risk of missing the Graf type IIa and IIb hips is considerably high by performing clinical examination alone [39]. The results of a meta-analysis revealed that there was still no strong evidence for the diagnostic accuracy of hip ultrasonography as a screening tool [40]. It is the author’s opinion that ultrasonographic hip screening is better than clinical hip screening alone, even if the clinical examination is performed by an experienced physician.

The exact answer to the question concerning the superiority of universal or selective hip screening programmes is still debatable. There exists conflicting evidence that universal screening significantly increases the rate of treatment [33, 40]. A nationwide universal ultrasonographic newborn hip screening has been carried out in Austria and Germany since 1992 and 1996, respectively [7]. The previously reported experiences from these countries seem to be promising. The rate of open reduction in late diagnosed cases was 0.35/1,000 live births in 1992 and 0.13/1,000 live births in 2004 in Austria. Besides, the rate of surgical intervention including pelvic osteotomy and acetabuloplasty until 2 years of age decreased from 3.5/1,000 live births in 1992 to 0.24/1,000 live births in 2004. The treatment costs in DDH were reduced to about 80 % when compared with the pre-screening period [7]. The results of another study from Austria revealed that universal hip screening significantly reduced the initial treatment age, made more simple treatment methods available, shortened the treatment time and decreased the rate of avascular necrosis of the femoral head following primary treatment in the unstable hips [41]. Universal screening was found to decrease the rate of first operative procedures, including closed or open reduction or osteotomy, to 52 % in a recent report from Germany [42]. In another study from Germany, it was noted that a universal screening programme reduced the rate of late detected DDH as well as the number of surgically treated hips [43]. On the other hand, the results of a 20-year-old selective hip screening programme revealed that the rate of late detected DDH was 0.34/1,000, and this programme was considered effective [44].

In a prospective randomised study, the rates of late detected subluxation/dislocation per 1,000 newborns were 0.3 and 0.7 in the universal and selective screening groups, respectively [37]. Among the ultrasonographically screened babies in this study, about 15 % were re-examined at maturity, and universal and selective screening groups were found to have similar radiographic findings concerning early degenerative changes, acetabular slope and femoral head coverage [38]. In another prospective randomised study, the rate of late detected DDH per 1,000 live births was found to be 0.13 in the universal screening group and 0.65 in the selective screening group [36]. Nevertheless, there is still no strong evidence to support the superiority of either universal or selective ultrasonographic hip screening in avoiding the late detected cases and lessening the rate of surgical procedures, as the power of the previous studies has not been considerably high [33]. It is the author’s opinion that the selection of the type of the national or local ultrasonographic hip screening programme depends on several factors, such as the status of the national health-care system, number of live births per year, number of educated health-care professionals taking part in the hip screening programme, parents’ sensitivity about the hip screening programme etc.

The optimal time for ultrasonographic screening is also controversial. The results of an ultrasonographic study revealed that, among the Graf type IIa or worse hips which were determined within the first 3 days of life, only 9 % would remain abnormal and require treatment during the follow-up period [45]. In another study, it was shown that, among the Graf type I hips at 1 month of age, 99.6 % would still be type I at 3 months of age [46]. The recommended ultrasonographic examination time of the newborns with clinically unstable hips or newborns with risk factors such as family history or breech presentation is within the first week of life and the recommended ultrasonographic examination time of the remaining newborns is between 4 and 6 weeks of age [47]. We have been performing ultrasonographic hip screening for all newborns at 3–4 weeks of age and our results revealed that the rate of closed or open reduction due to failed conservative treatment was 7 % and the rate of osteotomy was 0 % after starting the institutional ultrasonographic hip screening programme [48].

Ultrasonography is also useful for monitoring conservative treatment, including the Pavlik harness [2].

## Conclusion

Clinical examination still has diagnostic value in newborn hip screening, principally in highly experienced hands. However, hip ultrasonography is currently the most accurate diagnostic tool in developmental DDH during early infancy. Besides, either the universal or the selective ultrasonographic newborn hip screening programmes have notably decreased the rate of late detected and surgically treated DDH cases. It is better to perform the ultrasonographic hip screening within the first month of life. An effective hip ultrasonography method should include simple, precise, quantitative and consistent definitions for obtaining accurate diagnosis and managing the hip dysplasia in a proper way. So, the Graf method seems to meet all these mentioned requirements for accurate identification of the hip morphology, as well as proper management of the newborn hip joint. It can be better to finish this review article using a motto from Reinhard Graf “better ultrasound today than a limp tomorrow” [4] and another motto from Alain Dimeglio from Montpellier, France “prevention is winning wars elegantly without bloodshed” [49].
